# Tsetse Immune System Maturation Requires the Presence of Obligate Symbionts in Larvae

**DOI:** 10.1371/journal.pbio.1000619

**Published:** 2011-05-31

**Authors:** Brian L. Weiss, Jingwen Wang, Serap Aksoy

**Affiliations:** Department of Epidemiology and Public Health, Division of Epidemiology of Microbial Diseases, Yale University School of Medicine, New Haven, Connecticut, United States of America; Stanford University, United States of America

## Abstract

Beneficial microbial symbionts serve important functions within their hosts, including dietary supplementation and maintenance of immune system homeostasis. Little is known about the mechanisms that enable these bacteria to induce specific host phenotypes during development and into adulthood. Here we used the tsetse fly, *Glossina morsitans*, and its obligate mutualist, *Wigglesworthia glossinidia*, to investigate the co-evolutionary adaptations that influence the development of host physiological processes. *Wigglesworthia* is maternally transmitted to tsetse's intrauterine larvae through milk gland secretions. We can produce flies that lack *Wigglesworthia* (*Gmm^Wgm^* ^−^) yet retain their other symbiotic microbes. Such offspring give rise to adults that exhibit a largely normal phenotype, with the exception being that they are reproductively sterile. Our results indicate that when reared under normal environmental conditions *Gmm^Wgm^* ^−^ adults are also immuno-compromised and highly susceptible to hemocoelic *E. coli* infections while age-matched wild-type individuals are refractory. Adults that lack *Wigglesworthia* during larval development exhibit exceptionally compromised cellular and humoral immune responses following microbial challenge, including reduced expression of genes that encode antimicrobial peptides (*cecropin* and *attacin*), hemocyte-mediated processes (*thioester-containing proteins 2* and *4* and *prophenoloxidase*), and signal-mediating molecules (*inducible nitric oxide synthase*). Furthermore, *Gmm^Wgm^* ^−^ adults harbor a reduced population of sessile and circulating hemocytes, a phenomenon that likely results from a significant decrease in larval expression of *serpent* and *lozenge*, both of which are associated with the process of early hemocyte differentiation. Our results demonstrate that *Wigglesworthia* must be present during the development of immature progeny in order for the immune system to function properly in adult tsetse. This phenomenon provides evidence of yet another important physiological adaptation that further anchors the obligate symbiosis between tsetse and *Wigglesworthia*.

## Abstract 2

Tsetse harbors an obligate symbiont, *Wigglesworthia glossinidia,* that must be present during larval maturation for the fly's immune system to develop and function properly during adulthood.

## Author Summary

Beneficial bacterial symbionts, which are ubiquitous in nature, are often characterized by the extent to which they interact with the host. In the case of mutualistic symbioses, both partners benefit so that each one can inhabit diverse ecological niches where neither could survive on its own. Unfortunately, little is known about the functional mechanisms that underlie mutualistic relationships. Insects represent a group of advanced multi-cellular organisms that harbor well-documented symbiotic associations. One such insect, the tsetse fly, harbors a maternally transmitted bacterial mutualist called *Wigglesworthia* that provides its host with essential metabolites missing from its vertebrate blood-specific diet. In this study, we further examine the relationship between tsetse and *Wigglesworthia* by investigating the interaction between this bacterium and its host's immune system. We have found that when *Wigglesworthia* is absent from tsetse during the maturation of immature larval stages, subsequent adults are characterized by an underdeveloped cellular immune system and thus highly susceptible to infection with a normally non-pathogenic foreign microbe. These findings represent an additional adaptation that further anchors the steadfast relationship shared between tsetse and its obligate symbiont.

## Introduction

Bacteria comprise the most abundant and diverse life form on earth. The ubiquity of bacteria means they have colonized virtually every ecological niche, including habitation within more evolutionarily sophisticated multi-cellular animals. Co-evolution over millions of years has provided an opportunity for beneficial symbiotic associations to develop between phylogenetically distant taxa. Such affiliations are often mutualistic, meaning both partners benefit so that each can successfully inhabit diverse environments that neither could survive in on its own [1],[2]. Deciphering the mutualistic relationships between prokaryotic bacteria and multi-cellular eukaryotic animals is a rapidly advancing field of research.

Performing detailed investigations into the relationships between symbiotic bacteria and higher eukaryotes often involves costly and complex procedures. However, insects have well-documented symbioses that are attractive to study because they have relatively short generation times and are easy and less costly to rear. One insect that harbors multiple symbionts is the tsetse fly, *Glossina morsitans*. These microbes include the commensal *Sodalis*, the parasite *Wolbachia*, and the obligate mutualist *Wigglesworthia glossinidia* [3]. Molecular phylogenetic analysis indicates that tsetse's symbiosis with *Wigglesworthia* is ancient, dating back 50–80 million years [4]. The concordant nature of tsetse's obligate association with *Wigglesworthia* has driven the co-evolution of biological adaptations that are beneficial to both partners. For example, the localization of *Wigglesworthia* cells within host bacteriocytes provides a protective and metabolically favorable niche for this bacterium [5]. In return tsetse derives benefit from *Wigglesworthia* in at least two distinct ways. First, tsetse feeds exclusively on vertebrate blood, which is deficient in vitamins essential for survival. In accordance, a large proportion of *Wigglesworthia's* streamlined (700 kB) genome encodes vitamin biosynthesis pathways that presumably supplement tsetse's restricted diet [5],[6]. Second, more recent studies indicate that *Wigglesworthia* may serve an immunologic role in tsetse. These flies are the sole vector of pathogenic African trypanosomes, the causative agent of sleeping sickness in humans [7]. In laboratory experiments infection with immunogenic trypanosomes results in a decrease in tsetse fecundity [8]. Furthermore, parasite infection prevalence is higher in flies that lack *Wigglesworthia* when compared to age-matched wild-type (WT) individuals [9]. *Wigglesworthia* is thought to influence tsetse's vectorial competence by modulating its host's humoral immune system [10]. Thus, by preventing energetically costly parasite infections, this obligate symbiont may indirectly benefit the reproductive fitness of tsetse.

Symbiotic bacteria are rapidly gaining recognition for their important contributions to host development and immunity. The most well-known example of this type of interaction involves the mammalian microbiome, which modulates gut development during early postnatal life and subsequently shapes our mucosal and systemic immune systems [11],[12]. Symbionts also serve similar functions in invertebrate hosts. For example, light organ morphogenesis in juvenile bobtail squid (*Euprymna scolopes*) initiates only after symbiotic *Vibrio fischeri* cells have stably colonized this tissue [13]. The pea aphid, *Acyrthosiphon pisum*, harbors a secondary symbiont, *Hamiltonella defensa*, which can be infected with a lysogenic bacteriophage (*A. pisum* secondary endosymbiont; APSE). *A. pisum* that harbor both *H. defensa* and APSE are protected from being consumed by a parasitic wasp larvae through the action of phage-encoded toxins [14].

To the best of our knowledge no evidence exists that demonstrates insect symbionts can confer an impending protective phenotype in their adult host by directing immune system development during immature stages. In the present study we investigate the mechanism by which *Wigglesworthia* contributes to the development and function of cellular and humoral immune responses in adult tsetse. Our results show that the maturation and normal function of adult cellular immune responses in tsetse are severely compromised when *Wigglesworthia* is absent during larval development. This study reveals an important new facet that further anchors the obligate relationship between tsetse and *Wigglesworthia* and may serve as a useful model to understand the highly integrated and dynamic relationship between hosts and their beneficial bacterial fauna.

## Results

### Tsetse's Susceptibility to *E. coli* Infection Is Dependent Upon Age and Symbiotic Status

**Figure 1:**
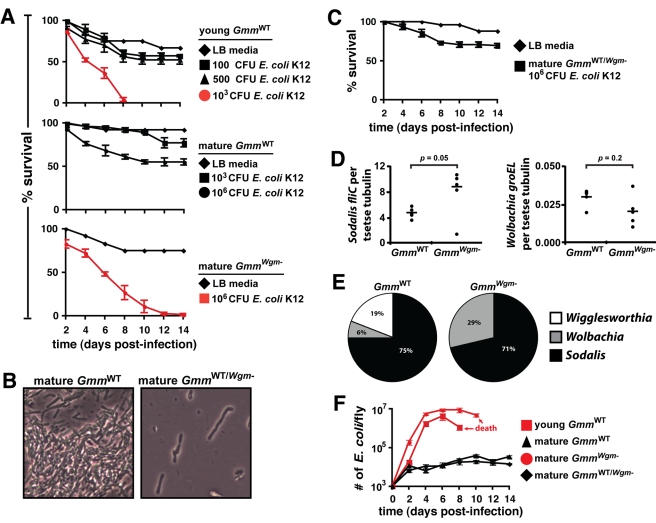
Host survival correlates with symbiont status following septic infection with *E. coli* K12. (A) The effects of age and symbiont status on the survival of tsetse following systemic infection with *E. coli* K12. Mature adult *Gmm^Wgm-^* flies were significantly more susceptible to infection with 10^6^ CFU of *E. coli* than were their wild-type counterparts (bottom and middle panels; *p*<0.001). (B) *Gmm* ^WT/*Wgm*−^ flies harbored *Wigglesworthia* during immature development but lacked the bacteria as mature adults. (C) *Gmm* ^WT/*Wgm*−^ adults were infected with tetracycline resistant *E. coli* 1 d after their last antibiotic-supplemented blood meal. Unlike their counterparts that lacked *Wigglesworthia* throughout immature development, *Gmm* ^WT/*Wgm*−^ was able to survive infection with *E. coli*. No significant difference in survival was observed between mature adult *Gmm* ^WT^ versus *Gmm* ^WT/*Wgm*−^ adults infected with 10^6^ CFU of *E. coli* (Figure 1A middle panel and Figure 1C; *p*  = 0.07). (D) Relative *Sodalis* and *Wolbachia* densities in 40-d-old *Gmm* ^WT^ and *Gmm^Wgm^* ^−^ adults (*n*  = 5 of each) were normalized against host *β-tubulin* copy number. (E) Analysis of bacterial 16s rRNA clone libraries indicates that *Gmm* ^WT^ larvae harbored *Wigglesworthia*, *Sodalis*, and *Wolbachia*, while their counterparts from ampicillin treated females harbored only *Sodalis* and *Wolbachia*. No other bacteria were identified from either fly line. (F) Average number (±SEM) of rec*E. coli*_pIL_ per tsetse strain over time (*n*  = 3 individuals per strain per time point) following septic infection with 10^3^ CFU of bacteria. Values shown in red represent lethal infections.

Insects are normally capable of mounting an immune response that combats infection with various groups of bacteria. Interestingly, in comparison to *Drosophila*, tsetse flies are uniquely susceptible to septic infection with 10^3^ colony-forming units (CFU) of normally non-pathogenic *Escherichia coli* (*E. coli*) K12 [15]. In the present study we further investigated tsetse's unique susceptibility to *E. coli* infection by subjecting wild-type (*Gmm* ^WT^) and adults from two age groups to hemocoelic infections with varying quantities of *E. coli* K12. Three-day-old *Gmm* ^WT^ individuals (flies from this age group are hereafter referred to as “young”) were highly susceptible to this treatment, as 10^3^ CFU resulted in the death of all flies by 8 d post-infection (dpi; Figure 1A, top graph). In contrast, 77% and 55% of 8-d-old WT individuals (flies 8 d old and older are hereafter referred to as “mature”) survived for 14 dpi with 10^3^ and 10^6^ CFU of *E. coli* K12, respectively (Figure 1A, middle graph).

We demonstrated previously that feeding pregnant female tsetse the antibiotic ampicillin results in the generation offspring that lack *Wigglesworthia* (*Gmm^Wgm^* ^−^) but still harbor *Sodalis* [9] and presumably *Wolbachia*. In the present study we determined the survival outcome of mature *Gmm^Wgm^* ^−^ inoculated with 10^6^ CFU of *E. coli* K12 (the dose required to kill ∼50% of mature WT flies; Figure 1A, middle graph). In comparison to age-matched WT tsetse, *Gmm^Wgm^* ^−^ flies were highly susceptible to this infection. In fact, at 14 dpi, only 1% of these individuals remained (Figure 1A, bottom graph). We reasoned that the dramatic susceptibility of mature *Gmm^Wgm^* ^−^ flies to infection with *E. coli* could result from one of two scenarios. In the first scenario *Wigglesworthia* may directly contribute to the adult immune response to a foreign micro-organism. In the second scenario, the absence of *Wigglesworthia* during larvagensis may give rise to *Gmm^Wgm^* ^−^ adults with compromised immune functions. To determine if the first scenario is correct we treated mature WT tsetse with the antibiotic tetracycline to eliminate all of their microbiota, including bacteriome-associated *Wigglesworthia* (Figure 1B), and subsequently challenged these adult flies (*Gmm* ^WT/*Wgm*−^) with 10^6^ CFU of tetracycline resistant *E. coli* K12. We found that, similar to their WT counterparts (*Gmm* ^WT^; Figure 1A, middle panel), about 70% of *Gmm* ^WT/*Wgm*−^ individuals survived this infection (Figure 1C). Conversely, *Gmm^Wgm^* ^−^ adults were highly susceptible (Figure 1A, bottom graph). This result suggests that when *Wigglesworthia* is absent from tsetse during larval development subsequent adults are severely immuno-compromised.

We demonstrated that when larval tsetse lacks exposure to *Wigglesworthia* in utero their immune system is highly compromised during adulthood. This finding does not exclude the possibility that treatment of female flies with ampicillin to produce *Gmm^Wgm^* ^−^ offspring induces perturbations in other constituents of tsetse's microbiota. Because tsetse's other two known endosymbionts, *Sodalis* and *Wolbachia*, are present at similar densities in *Gmm* ^WT^ and *Gmm^Wgm^* ^−^ adults (Figure 1D), we looked for the presence of uncharacterized endosymbionts or digestive tract-associated microbes that are potentially passed on to developing intra-uterine larvae where they subsequently impact immunity. We generated a clone library containing 16s rRNA gene sequences PCR amplified from 3^rd^ instar *Gmm^Wgm^* ^−^ and *Gmm* ^WT^ larvae and then sequenced multiple clones from each tsetse line. Our results indicate that the proportion of *Wigglesworthia*, *Sodalis*, and *Wolbachia* in 3^rd^ instar *Gmm* ^WT^ larvae is 19%, 75%, and 6%, respectively. As expected no *Wigglesworthia* 16s rRNA sequences were present in *Gmm^Wgm^* ^−^ larvae, and the proportion of *Sodalis* and *Wolbachia* sequences present was 71% and 29%, respectively (Figure 1E). No uncharacterized endosymbionts or gut-associated environmental microbes were present in any sample. Taken together, these results suggest that the presence of *Wigglesworthia* specifically is responsible for enabling immune system maturation in WT tsetse.

Our host survival curves indicate that mature *Gmm* ^WT^ and *Gmm* ^WT/*Wgm*−^ can survive infection with *E. coli* while young *Gmm* ^WT^ and mature *Gmm^Wgm^* ^−^ perish. To determine a cause for the variation in survival we observed between the four groups following infection with *E. coli*, we monitored bacterial growth dynamics over the course of the experiment in each group. Bacterial number within mature *Gmm* ^WT^ and *Gmm* ^WT/*Wgm*−^ peaked at 3.8×10^4^ and 1.9×10^4^ *E. coli*, respectively, over the 2-wk period, indicating that these groups appeared able to control their infections. In contrast, *E. coli* increased exponentially in young *Gmm* ^WT^ and mature *Gmm^Wgm^* ^−^ and reached a maximum density at 6 dpi of 4.2×10^6^ and 8.8×10^6^, respectively (Figure 1F). These results implicate bacterial sepsis as the cause of high mortality observed in young *Gmm* ^WT^ and mature *Gmm^Wgm^* ^−^. All of the above-mentioned results taken together indicate that mature *Gmm* ^WT^ are considerably more resistant to infection with a foreign microbe than are their younger counterparts. Furthermore, tsetse's obligate mutualist, *Wigglesworthia*, must be present during the development of immature stages so that mature adults are able to overcome infection with *E. coli*.

### Expression of Immunity-Related Genes in Adult Tsetse Varies Depending on Symbiont Status

To understand the basis of the compromised immunity we observed in mature *Gmm^Wgm^* ^−^ flies, we evaluated the expression profile of a set of immunity-related genes from age-matched mature adult *Gmm* ^WT^ and *Gmm^Wgm^* ^−^ flies that were either uninfected or 3 dpi with *E. coli* K12. We included the antimicrobial peptides (AMPs) *attacin*, *cecropin*, and *defensin*, which distinctly target gram-negative (*attacin* and *cecropin*) and gram-positive bacteria (*defensin*) [16]. Our analysis also included thioester-containing proteins (*tep*2 and *tep4*), prophenoloxidase (*PPO*), and inducible nitric oxide synthase (*iNOS*). In insects TEPs likely function as pathogen-specific opsonins that bind to bacteria or parasites and promote their phagocytosis/encapsulation [17], while PPO initiates a proteolytic cascade that results in melanin deposition [18]. iNOS catalyzes synthesis of the signaling molecule nitric oxide (NO), which plays a role in humoral and cellular immunity in Anopheline mosquitoes [19], reduvid bugs [20], and *Drosophila* [21],[22] by inducing AMP expression and recruiting hemocytes to the site of infection.

**Figure 2:**
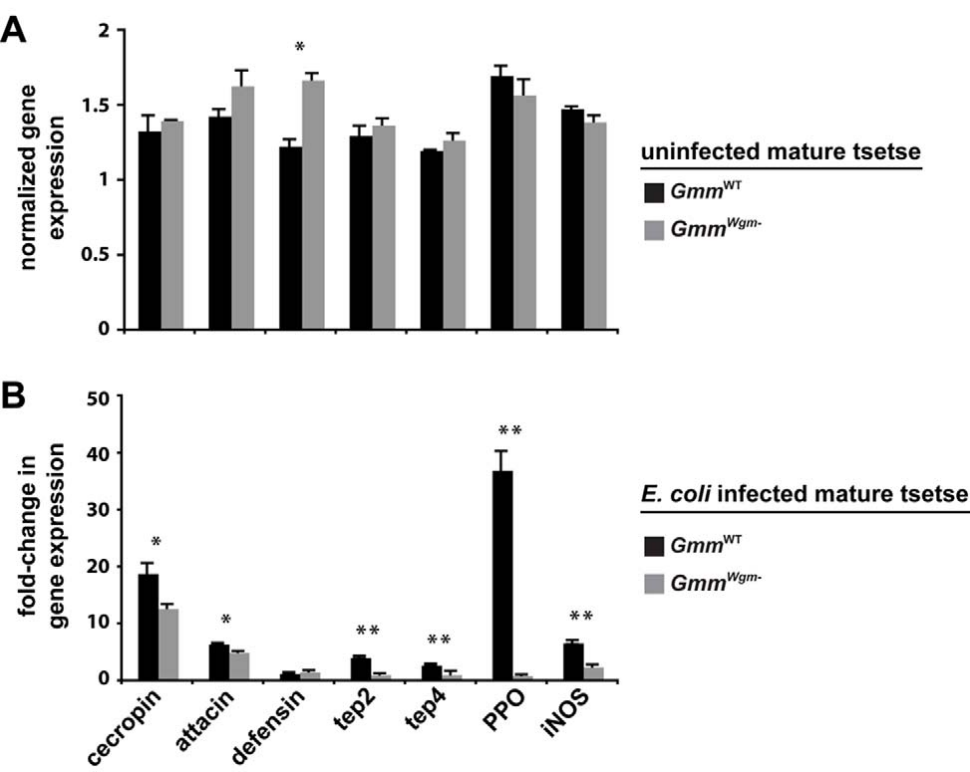
The effect of symbiont status on the expression of immunity-related genes in adult tsetse. (A) Target gene expression in uninfected *Gmm* ^WT^ and *Gmm^Wgm^* ^−^ adults is indicated relative to the constitutively expressed tsetse β-tubulin gene. (B) Fold-change in the expression of immunity-related genes in *Gmm* ^WT^ and *Gmm^Wgm^* ^−^ tsetse 3 dpi with *E. coli* K12. All values for both tsetse strains are represented as a fraction of average normalized gene expression levels in bacteria-infected flies relative to expression levels in PBS-injected controls. In (A) and (B), quantitative measurements were performed on three biological samples in duplicate. Values are represented as means (±SEM). * *p*<0.05, ** *p*<0.005 (Student's *t*-test).

Our expression results indicate that symbiont status plays little or no role in the expression of immunity-related genes in uninfected adults. In fact, with the exception of the AMP *defensin*, no significant differences in immunity-related gene expression between mature uninfected *Gmm* ^WT^ and *Gmm^Wgm^* ^−^ adults (Figure 2A). However, we observed a considerably different profile of immunity-related gene expression when these different fly strains were infected with *E. coli* K12. Under these circumstances, all of the genes evaluated (with the exception of defensin) were expressed at significantly higher levels in *Gmm* ^WT^ compared to *Gmm^Wgm^* ^−^ individuals (Figure 2B). Particular striking was the fact that the induction of pathways associated with cellular immunity, such as pathogen recognition (*tep*2 and *tep*4) and melanization (*PPO*), were significantly compromised in mature *Gmm^Wgm^* ^−^ adults. The absence of a robust cellular immune response is likely the cause of high mortality among these individuals following *E. coli* infection. This analysis indicates that *Wigglesworthia* must be present during the development of immature tsetse in order for immune-related genes to subsequently be expressed in mature *E. coli*–infected adults.

### Hemocytes Play an Integral Role in Controlling *E. coli* Infection Outcomes in Tsetse

Our analysis of immunity-related gene expression in tsetse suggests that cellular immune pathway functions in adult tsetse are particularly compromised when *Wigglesworthia* is absent during immature development. The most prominent cellular immune mechanisms include melanization and phagocytosis. These processes, which ultimately result in the removal of foreign invaders in *Drosophila* [23] and *A. aegypti* [24], both arise from distinct crystal cell and plasmatocyte hemocyte lineages, respectively [25].

**Figure 3:**
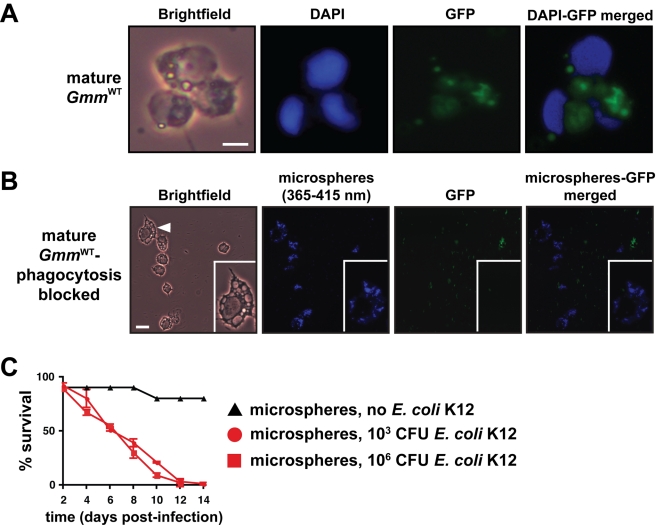
Hemocyte-mediated phagocytosis is a critical component of tsetse's immune response. (A) 8-d-old *Gmm* ^WT^ were subjected to septic infection with GFP-expressing *E. coli* K12. Twelve hours post-infection hemolymph was collected, fixed on glass slides using 2% paraformaldehyde, and microscopically examined for the presence of hemocyte-engulfed bacterial cells. Scale bar  = 10 µm. (B) The process of hemocyte-mediated phagocytosis in tsetse was blocked by micro-injecting polystyrene beads into the hemocoel of 8-d-old WT individuals. In consecutive 12 h intervals following bead injection, flies were infected with GFP-expressing *E. coli* K12 and then hemolymph was collected and fixed as described above. Hemocytes appear to have engulfed the beads, thus prohibiting the subsequent uptake of bacterial cells. The inset in each panel shows a higher magnification image of one hemocyte, which is identified by a white triangle in the left-most panel. Scale bar = 20 μm. (C) Tsetse flies that harbor hemocytes incapable of engulfing *E. coli* are susceptible to septic infection with this bacterium while their wild-type counterparts are not. The susceptible phenotype is exhibited regardless of whether phagocytosis-inhibited tsetse were inoculated with 10^3^ or 10^6^ CFU of *E. coli*. Beads alone had no effect on tsetse mortality. No significant difference existed in survival outcome between mature *Gmm* ^WT^ phagocytosis inhibited flies infected with 10^3^ versus 10^6^ CFU of *E. coli* (*p*  = 0.47, log-rank analysis). Furthermore, no significant difference was present between mature *Gmm^Wgm^* ^−^ flies with uninhibited hemocytes (Figure 1A, bottom panel) and mature *Gmm* ^WT^ phagocytosis inhibited flies (*p*  = 0.11) infected with 10^6^ CFU of *E. coli*.

We investigated the role hemocytes might play in determining the susceptible phenotype we observed in tsetse following infection with *E. coli*. We infected mature *Gmm* ^WT^ individuals with GFP-expressing *E. coli* and were able to observe that hemocytes had engulfed a large number of the introduced cells by 12 hpi (Figure 3A). We next inhibited phagocytosis by introducing blue fluorescent microspheres directly into tsetse's hemocoel and 12 h later infected the bead-treated individuals with GFP-expressing *E. coli*. Microscopic inspection of hemocytes harvested 12 hpi with *E. coli* revealed the presence of internalized microspheres and the absence of engulfed *E. coli*. This observation indicated that we were successful in blocking hemocyte phagocytosis (Figure 3B). We subsequently maintained our microsphere-injected tsetse for 2 wk with the intention of determining the impact of impaired phagocytosis on host survival outcome. Mature *Gmm* ^WT^ flies exhibiting impaired phagocytosis were highly susceptible to infection with both 1×10^3^ and 1×10^6^ *E. coli* K12. In fact, by day 12 post-infection, all of these flies had perished regardless of the initial dose used for infection (Figure 3C). This observation contrasts starkly with infection outcome in mature *Gmm* ^WT^ that exhibit normally functioning hemocytes (Figure 1A, middle graph). Our results suggest that defects in phagocytosis severely compromise the ability of these tsetse flies to overcome bacterial infection.

**Figure 4:**
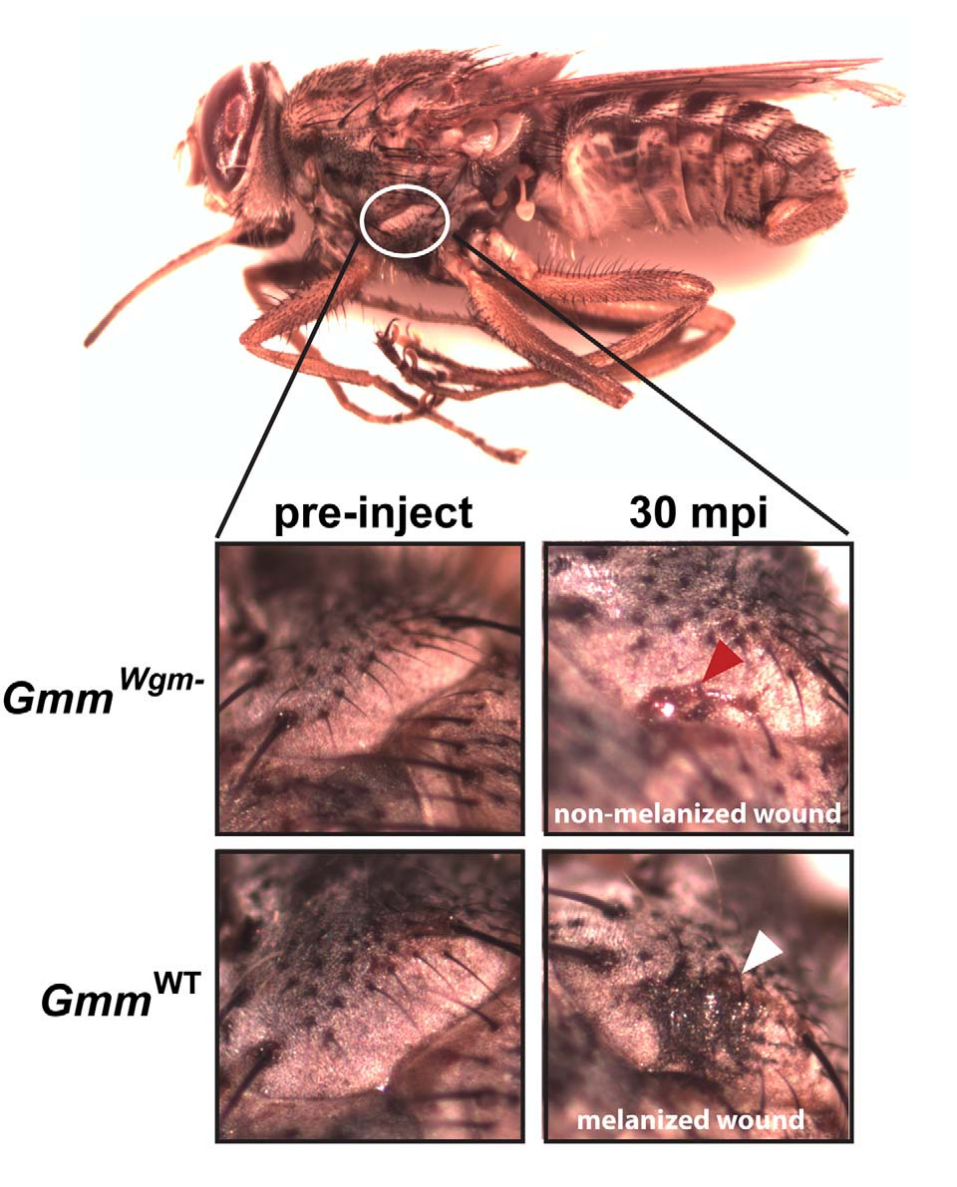
The effect of symbiont status on melanization in tsetse. Mature *Gmm* ^WT^ and *Gmm^Wgm^* ^−^ tsetse (*n*  = 10 of each strain) were intra-thoracically inoculated with 1×10^3^ *E. coli* K12. Thirty minutes post-inoculation the wound site on individuals from each strain was inspected microscopically for the presence of hemolymph clotting and melanin deposition. Thirty minutes post-inoculation, neither hemolymph clotting nor melanin were observed at the wound site of *Gmm^Wgm^* ^−^ individuals (indicated by a red arrow). In contrast, within the same amount of time, hemolymph no longer exuded from the wound of WT flies and melanin was present surrounding the site (indicted by a white arrow).

A notable result of our immunity-related gene expression analysis was a 37-fold decrease in *PPO* levels in *Gmm^Wgm^* ^−^ flies. This enzyme is an essential component of the melanization pathway, and its expression ultimately results in host wound healing and the melanization, encapsulation, and subsequent removal of foreign microorganisms [26]–[28]. In conjunction with the remarkable variation in *PPO* expression observed between *Gmm* ^WT^ and *Gmm^Wgm^* ^−^, we were also able to visually observe the absence of a melanization response to *E. coli* infection in flies lacking *Wigglesworthia*. In fact, 30 min post-injection with *E. coli*, hemolymph was still actively exuding from the inoculation wound of *Gmm^Wgm^* ^−^ flies. Conversely, in WT individuals no hemolymph was detectable and melanin was deposited at the wound site (Figure 4). These results further suggest that hemocyte-mediated cellular immunity provides an imperative defense against the establishment of bacterial infections in tsetse's hemocoel. Furthermore, the absence of this response in *Gmm^Wgm^* ^−^ individuals was likely responsible for the compromised host survival phenotype we observed following infection with *E. coli*.

### Symbiotic Bacteria Impact the Maturation of Tsetse's Cellular Immune Response During Development

**Figure 5:**
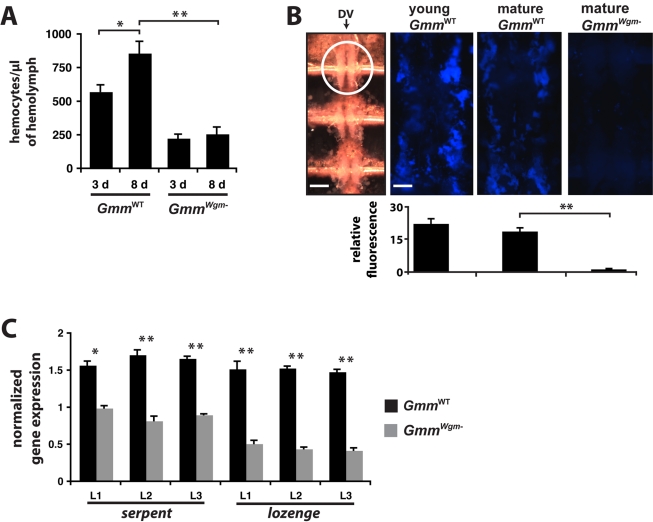
The effect of age and symbiont status on the development of tsetse's cellular immune response. (A) Number of hemocytes per µl of hemolymph in young (3 d) and mature (8 d) *Gmm* ^WT^ and *Gmm^Wgm^* ^-^ tsetse (*n* = 5 individuals from each tsetse strain at both time points). (B) Quantitative analysis of sessile hemocyte abundance in young and mature *Gmm* ^WT^ and mature *Gmm^Wgm^* ^-^ tsetse (*n* = 4 individuals from each tsetse strain and age point). All tsetse strains tested were subjected to hemocoelic injection with blue fluorescent microspheres. Twelve hours post-injection, flies were dissected to reveal tsetse's heart. The left-most panel is a Brightfield image of the three chambers that make up the dorsal vessel (DV; scale bar = 350 µm). The anterior-most chamber is indicated within a white circle. The three remaining panels are close-ups of the anterior chamber (scale bar for all 3 panels  = 80 µm), visualized by excitation with UV light (365/415 nm). Relative fluorescence per tsetse group was determined using ImageJ software. (C) The presence of *Wigglesworthia* affects the expression of genes involved in hemocyte differentiation in immature larval tsetse. Target gene expression in *Gmm* ^WT^ and *Gmm^Wgm^* ^−^ larval instars 1–3 is indicated relative to the constitutively expressed tsetse β-tubulin gene. Quantitative measurements were performed on three biological samples in duplicate. All values in this figure are represented as means (±SEM). * *p*<0.05, ** *p*<0.005 (Student' *t*-test).

We observed that young *Gmm* ^WT^ were markedly more susceptible to infection with *E. coli* K12 than were their mature counterparts. Furthermore, symbiont status also altered infection outcome, as mature *Gmm^Wgm^* ^−^ perished following *E. coli* infection while age-matched WT individuals survived. These differential infection outcomes appeared to result from disparities in cellular immune system function between the different tsetse lines we examined. Based on these observations we hypothesized that the obligate mutualist *Wigglesworthia* plays a crucial role in regulating the development of cellular immunity in tsetse during immature stages. To test this hypothesis we quantified the number of circulating and sessile hemocytes present in young and mature adult *Gmm* ^WT^ and mature adult *Gmm^Wgm^* ^−^. Our results indicate that a 1.4-fold increase in circulating hemocyte number occurs between day 3 and day 8 in WT tsetse, while no significant change in circulating hemocyte number was observed between young and mature *Gmm^Wgm^* ^−^ (Figure 5A). Interestingly, mature *Gmm* ^WT^ adults harbored 3.4× more circulating hemocytes than did mature *Gmm^Wgm^* ^−^ adults (Figure 5A). We also looked at sessile hemocyte abundance as a further indicator of *Wigglesworthia's* impact on the development of cellular immunity in tsetse. In *Drosophila*, this hemocyte subtype concentrates in large quantities around the anterior end of the fly's dorsal vessel [29]. Thus, we indirectly quantified sessile hemocyte abundance immediately adjacent to the anterior-most chamber of tsetse's dorsal vessel by measuring the fluorescent emission of microspheres that were found engulfed in this region. Young *Gmm* ^WT^ adults engulfed 1.2× more microspheres than their mature counterparts and 15.7× more than mature *Gmm^Wgm^* ^−^ adults. Furthermore, mature WT adults engulfed 13.2× more microspheres than did age-matched *Gmm^Wgm^* ^−^ adults (Figure 5B).

Our results demonstrate that *Wigglesworthia* must be present during the development of immature tsetse in order for cellular immunity to develop and function properly in adults. Thus, we speculate that the absence of hemocytes in adult tsetse should reflect a lack of blood cell differentiation during the development of immature stages. In *Drosophila* the process of blood cell differentiation, or hematopoiesis, begins in the embryo and proceeds through all larval stages [30]. During *Drosophila* embryogenesis early hematopoiesis can be distinguished by the expression of the zinc finger transcription factor “Serpent.” Subsequently, another transcription factor, “Lozenge,” directs the differentiation of *serpent*-expressing precursor cells into a specific lineage of hemocytes called crystal cells. To address the relationship between the presence of *Wigglesworthia* and early hematopoiesis in tsetse, we used qPCR to evaluate the relative number of *serpent* and *lozenge* transcripts present in 1^st^, 2^nd^, and 3^rd^ instar larvae dissected from pregnant *Gmm* ^WT^ and *Gmm^Wgm^* ^−^ females. Larval instars L1, L2, and L3 from WT females expressed 1.7, 2.1, and 1.9 times more *serpent* transcripts, and 4, 4.4, and 3.9 times more *lozenge* transcripts, respectively, than did their counterparts from females that lacked *Wigglesworthia* (Figure 5C). This observed attenuated expression of both *serpent* and *lozenge* may account for the depleted hemocyte population, and compromised cellular immune function, we observed in adult *Gmm^Wgm^* ^−^ individuals.

## Discussion

**Figure 6:**
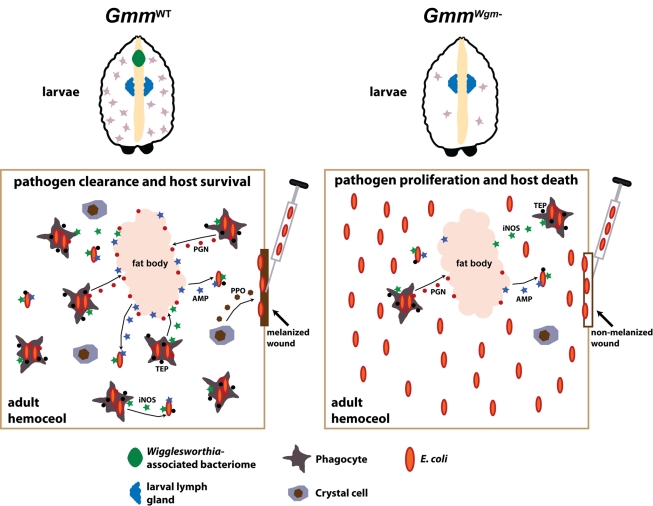
*Wigglesworthia* modulates the development and function of tsetse's immune system. Through a currently unknown mechanism, the presence of *Wigglesworthia* in *Gmm* ^WT^ larvae stimulates hemocyte differentiation in a specialized organ that is homologous to *Drosophila's* lymph gland. Upon metamorphosis, specialized hemocyte subtypes are released from the lymph gland and carried over in a functional state to the adult. In the absence of *Wigglesworthia*, *Gmm^Wgm^* ^−^ larvae produce significantly less hemocytes than their WT counterparts. Several innate immunity pathways are activated upon inoculation of *E. coli* into the hemocoel of mature adult *Gmm* ^WT^. Tsetse's preliminary line of defense against *E. coli* infection likely involves melanization at the wound site. This process is initiated by localized crystal cells, which instigate the melanization cascade by secreting prophenoloxidase (PPO) into the hemolymph. Pathogens that circumvent the wound site then encounter phagocyte-mediated cellular and humoral immune responses. Soluble TEPs likely opsonize bacterial cells, thus tagging them for engulfment by phagocytes. Lysis of engulfed bacteria causes the release of bacterial peptidoglycan PGN that subsequently stimulates the production of AMPs by the fat body. AMPs, which are also generated by hemocytes, are then secreted into the hemolymph where they further abrogate microbial proliferation. Hemocytes also produce reactive oxygen intermediates, such as iNOS, that exhibit direct bacterial toxicity and further stimulate humoral immunity. In the case of *Gmm^Wgm^* ^−^ adults, incapacitated hematopoiesis during larval stages results in severely compromised immunity that renders these flies highly susceptible as adults to bacterial infection.

In the present study we demonstrate that *Wigglesworthia* is intimately involved in regulating the maturation and function of tsetse's cellular immune system during immature larval development. We present a model that links the presence of *Wigglesworthia* in larval progeny with host immune system maturation during development and the subsequent ability of adult tsetse to overcome infection with foreign microbes (Figure 6). Obligate symbioses between intracellular bacteria and multi-cellular eukaryotes represent millions of years of co-evolution during which time both partners have adapted to increase each other's overall fitness. The association between tsetse and *Wigglesworthia* is an example of this reciprocal relationship in that neither organism can survive in the absence of the other.

We present several lines of evidence indicating that tsetse's resistance to *E. coli* positively correlates with fly age and symbiont infection status during juvenile stages. Specifically, our results signify that mature *Gmm* ^WT^ adults are resistant to *E. coli* infection, while young *Gmm* ^WT^ adults are susceptible. In contrast, both young and old adult flies that lack *Wigglesworthia* throughout all developmental stages are killed by *E. coli* infections. Like their WT counterparts, *Gmm^Wgm^* ^−^ larvae acquire both *Sodalis* and *Wolbachia* while in utero [31]. Although the intrauterine larval environment is otherwise aseptic, adult *Gmm^Wgm^* ^−^ can be exposed to a wide range of environmental microbes during adulthood. However, neither the presence of other symbiotic bacteria (*Sodalis* and *Wolbachia*) in larvae, nor environmental microbes acquired during adulthood, appear to be sufficient to induce immunity in *Gmm^Wgm^* ^−^ adults. Additionally, we show that mature *Gmm* ^WT^ adults treated with antibiotics to eliminate *Wigglesworthia* (*Gmm* ^WT/*Wgm*−^) remain resistant to *E. coli* infections. The resistant phenotype of *Gmm* ^WT/*Wgm*−^ further signifies that obligate *Wigglesworthia* is not directly responsible for the ability of mature adult *Gmm* ^WT^ to overcome septic bacterial infection. Instead, it appears that *Wigglesworthia*'s presence during the maturation of larval progeny stimulates the development of host immunity in adults.

Our quantitative analysis of gene expression indicates that *Gmm* ^WT^ and *Gmm^Wgm^* ^−^ adults exhibit no significant difference in the expression of genes that serve as hallmarks of humoral and cellular immunity in the absence of microbial challenge. However, following infection with *E. coli*, all pathways were significantly compromised in *Gmm^Wgm^* ^−^ versus *Gmm* ^WT^ adults. The most notable discrepancy observed between the two tsetse lines involved the expression of *prophenoloxidase*. Following *E. coli* infection, the expression of this gene increased 37-fold in WT tsetse but remained virtually unchanged in individuals that lacked *Wigglesworthia*. In WT insects, PPO, which is an inactive zymogen, is proteolytically cleaved to produce phenoloxidase upon mechanical injury or the presence of foreign pathogens. Phenoloxidase then facilitates the process of melanin deposition [18]. A functional melanotic pathway would likely increase tsetse's resistance to *E. coli* infection in several ways. First, sequestration of *E. coli* in a melanotic capsule at the site of inoculation would likely help prevent their dissemination into adjacent host tissues. This phenomenon has been observed in both the hawk moth, *Manduca sexta*, and *Drosophila* following infection with pathogenic bacteria (*Photorhabdus luminescens*) and parasitic wasp eggs, respectively [32],[33]. In both cases the lack of melanization at the wound site severely compromised the host's ability to subsequently overcome the foreign invader. Second, the melanization cascade results in the production of reactive oxygen intermediates that are directly toxic to foreign pathogens. In the flesh fly *Sarcophaga peregrina* and *M. sexta*, melanization intermediates such as DOPA exhibit direct antimicrobial activity [34],[35]. Finally, melanin at the site of inoculation expedites wound healing that could prevent the spread of secondary infections [26].

The *E. coli*–susceptible phenotype of mature *Gmm^Wgm^* ^−^ adults is likely reflective of their blood cell (hemocyte) deficit in comparison to *E. coli*–resistant WT flies. In *Drosophila* 90%–95% of the total hemocyte population is composed of sessile and circulating plasmatocytes, which are a distinct hemocyte lineage predominantly responsible for engulfing and digesting foreign pathogens [36]. The inability of mature *Gmm^Wgm^* ^−^ tsetse to survive infection with *E. coli* may result from their significantly reduced population of phagocytic hemocytes available to engulf bacteria injected into the hemocoel. In fact, by injecting polystyrene beads as a means of blocking this physiological process, we demonstrate that phagocytosis is a critical component of tsetse's ability to manage septic infection with *E. coli* in WT flies. Similarly, mutant *Drosophila* that contain a depleted plasmatocyte population, or have been injected with beads to prohibit phagocytosis, exhibit a remarkable susceptibility to a variety of gram positive and negative bacteria [37],[38]. Interestingly, *Drosophila* that lack functional plasmatocytes also exhibit a reduced capacity to activate humoral immune responses, further inhibiting their ability to fight bacterial infection [29].

In *Drosophila* the crystal cell hemocyte lineage controls the humoral melanization cascade via the release of PPO stored in large cytoplasmic inclusion bodies [30],[39]. In addition to the dramatic reduction in *PPO* expression in *Gmm^Wgm^* ^−^ tsetse, two further lines of evidence indicate that this fly strain harbors a significantly reduced population of hemocytes that function in a homologous manner to *Drosophila* crystal cells. First, *lozenge* expression in all three larval instars from *Gmm^Wgm^* ^−^ females is significantly lower than in their *Gmm* ^WT^ counterparts. In *Drosophila* larval crystal cells fail to form in the absence of *lozenge* expression, yet the differentiation of other hemocyte types proceeds normally [40]. Second, we observed that hemocyte-deficient *Gmm^Wgm^* ^−^ flies were unable to produce a viable clot at the site of bacterial inoculation. In mutant *Drosophila* strains that lack crystal cells, PPO is absent from the hemolymph. Consequently, the melanization cascade fails to initiate and hard clots do not form at wound sites [41].

We discovered that young *Gmm* ^WT^ adults harbor significantly less circulating hemocytes than do mature WT adults. This observation implies that circulating hemocyte number in adult WT tsetse increases as a function of age, although the specific mechanism underlying this process in tsetse is currently unknown. In adult *Drosophila* intact lymph glands are absent and no evidence exists for the de novo synthesis of hemocytes following metamorphosis [30],[40]. Interestingly, we did observe more subepidermal sessile hemocytes in young compared to mature *Gmm* ^WT^ adults, although the number was not statistically significant between the two groups. We speculate that the increased abundance of circulating hemocytes we observed in mature compared to young *Gmm* ^WT^ may reflect a shift in the proportion of sessile to circulating cells instead of de novo production of new hemocytes. A comparable process occurs in *Drosophila* larvae and adults, where the proportion of sessile to circulating hemocytes changes following immune stimulation [42],[43]. Furthermore, after larval *Drosophila* receive an epidermal wound, circulating hemocytes are rapidly recruited to the site of injury. These circulating cells are phagocytically active and likely function as a front-line surveillance system against tissue damage and microbial infection [44]. In the mosquito malaria vector *Anopheles gambiae*, exposure to *Plasmodium* parasites stimulated an increase in the number of granulocytes circulating in the hemolymph. These primed mosquitoes were subsequently more resistant to infection with pathogenic bacteria than their wild-type counterparts [45]. We propose that young *Gmm* ^WT^ adults and mature *Gmm^Wig^* ^−^ adults may be susceptible to *E. coli* infection in part because they harbor significantly less circulating hemocytes than do older WT individuals that are resistant. Our previous studies indicated that *Gmm^Wig^* ^−^ adults are highly susceptible to trypanosome infection [9],[10] and that this phenotype may be modulated by tsetse's humoral immune system [46]–[48]. Studies are ongoing to determine if cellular immunity also modulates tsetse's trypanosome vectorial competence.

Neonatal humans (and presumably other mammals as well) acquire probiotic gut microbes through the act of breast feeding [49]. These bacteria subsequently stably colonize the naïve intestine where they promote immune system maturation and enhance defense against infection with pathogenic microbes [50]. Most lower eukaryotes, including insects, hatch from an egg deposited into the environment and thus rely mainly on environmental microbes to stimulate their innate immune systems during the development of immature stages. For example, *Anopheline* mosquitoes and *Drosophila* are exposed to environmental microbes throughout all stages of their development. Under these natural conditions subsequent adults exhibit potent innate immunity [51],[52]. However, when these insects are reared under germ-free conditions, they exhibit a severely compromised humoral immune response [51],[53]. Tsetse flies are unique among other insects because they lead a relatively aseptic existence. Not only do they feed exclusively on sterile vertebrate blood, but they also exhibit a unique viviparous reproductive strategy. During this type of reproduction, all embryonic and larval stages develop within the female's uterus. The protected in utero environment in which viviparous offspring develop limits their exposure to environmental microbes. However, throughout tsetse larvagenesis maternal milk gland secretions provide the developing offspring with nourishment as well as *Wigglesworthia*, *Sodalis*, and *Wolbachia* [31]. Interestingly, recently established *Sodalis* and *Wolbachia* appear unable to influence their host's physiology to the same extent as mutualist *Wigglesworthia* despite their presence throughout larval development. Future studies on this system will focus on determining the chemical and/or metabolic elements provided by *Wigglesworthia* that stimulate the maturation of its host's immune system during larval development.

The results of this study provide evidence of a novel functional role for obligate insect symbionts–host immune system activation during immature developmental stages to ensure robust function during adulthood. We demonstrate that the obligate *Wigglesworthia* provides tsetse offspring the stimuli necessary for immune system development, a process that exhibits functional parallels with the mammalian system following the transfer of beneficial microbes from mothers to their neonates during that act of breast feeding. This phenomenon represents an adaptation that further anchors the steadfast relationship shared between tsetse and its obligate mutualist. The essential nature of tsetse's dependence on *Wigglesworthia* provides a potentially exploitable niche to experimentally modulate host immunity, with the intention of diminishing this insect's capacity to vector deadly trypanosomes.

## Materials and Methods

### Tsetse and Septic Infections

Throughout the text, 3-d-old and 8-d-old tsetse are referred to as “young” and “mature,” respectively. Wild-type *G. m. morsitans* (*Gmm* ^WT^) were maintained in Yale's insectary at 24°C with 50%–55% relative humidity. These flies received defibrinated bovine blood every 48 h through an artificial membrane feeding system [54]. Two *Wigglesworthia*-free tsetse lines were generated for use in our experiments. The first, *Gmm^Wgm^* ^−^, was generated by supplementing the diet of pregnant females with 25 µg of ampicillin per ml of blood. *Gmm^Wgm^* ^−^ (offspring of ampicillin-fed females) adult are devoid of *Wigglesworthia* throughout all developmental stages [9]. PCR using *Wigglesworthia thiamine C-*specific primers (forward, 5′-TGAAAACATTTGCAAAATTTG-3′; reverse, 5′-GGTGTTACATAGCATAACAT-3′) confirmed that this tsetse strain lacked *Wigglesworthia.* The second *Wigglesworthia*-free tsetse line, *Gmm* ^WT/*Wgm*−^, was generated by feeding mature WT adults three blood meals supplemented with 40 µg/ml tetracycline. *Gmm* ^WT/*Wgm*−^ individuals thus harbored *Wigglesworthia*, *Sodalis*, and *Wolbachia* throughout the development of all immature stages and early adulthood, but lacked these symbionts thereafter. The absence of *Wigglesworthia* from mature *Gmm* ^WT/*Wgm*−^ adults was confirmed microscopically by comparing the bacteriome (*Wigglesworthia*-harboring organ) contents of mature WT and *Gmm* ^WT/*Wgm*−^ adults.

Septic infection of tsetse was achieved by anesthetizing flies with CO_2_ and subsequently injecting individuals with live bacterial cells using glass needles and a Narashige IM300 micro-injector. The methods used to produce luciferase-expressing *E. coli* K12 (rec*E. coli*_pIL_), and the assay used to quantify luciferase expression in vivo, were described previously [15]. GFP-expressing and tetracycline-resistant *E. coli* K12 were produced via electroporation with pGFP-UV (Clontech, Mountain View, CA) and pBR322 (Promega, Madison, WI) plasmid DNA, respectively. All flies treated with tetracycline were subsequently infected with tetracycline resistant *E. coli* K12. The number of bacterial cells injected and control group designations for all infection experiments are indicated in the corresponding figures and their legends. For all survival experiments, treatments were performed in triplicate, using 25 flies per *E. coli* treatment replicate. LB media controls were performed once using 25 flies.

### Real-Time Quantitative PCR (qPCR)

For analysis of immunity-related gene expression, sample preparation and qPCR were performed as described previously [15]. Quantitative measurements were performed on three biological samples in duplicate and results were normalized relative to tsetse's constitutively expressed β-tubulin gene (determined from each corresponding sample). Fold-change data are represented as a fraction of average normalized gene expression levels in bacteria-infected flies relative to expression levels in corresponding uninfected controls.

For symbiont quantification, total RNA was prepared from 40-d-old adult *Gmm* ^WT^ and *Gmm^Wgm^* ^−^ flies. Symbiont genome numbers were quantified using single-copy *Sodalis fliC* and *Wolbachia groEL*. Relative symbiont densities were normalized to tsetse *β-tubulin*. All qPCR was performed with an icycler iQ real time PCR detection system (Bio-Rad). Values are represented as the mean (±SEM). qPCR primer sequences are shown in Table S1.

### Bacterial 16s rRNA Clone Libraries

Universal bacterial 16S rRNA gene primers 27F (5′-AGAGTTTGATCCTGGCTCA G-3′) and 1492R (5′-GGTTACCTTGTTACGACTT-3′) [55] were used to produce a clone library of 16s rRNA gene sequences found in 3^rd^ instar *Gmm* ^WT^ and *Gmm^Wgm^* ^−^ larvae. Five individual larvae from each tsetse line were dissecting under sterile conditions and washed in DNase (Ambion, Austin, TX) to remove any surface contamination prior to DNA extraction. Genomic DNA was isolated using Holmes-Bonner buffer (0.1 mol/L Tris-HCl, pH 7.5; 0.35 mol/L NaCl; 10 mmol/L EDTA, pH 8.0; 2% SDS; 7 mol/L Urea), purified via phenol-chloroform extraction and precipitated in 100% EtOH. PCR was performed under standard reaction conditions [15], and the resulting products were cloned in the pGEM-T vector (Promega, Madison, WI). Twenty clone inserts from each larvae (100 in total from each tsetse line) were sequenced using the T7 vector specific primer, and homology to previously described 16s rRNAs was determined using the blastn database.

### Hemolymph Collection and Hemocyte Quantification

Depending on the subsequent experiment, tsetse hemolymph was collected using one of two methods. For hemocyte quantification, undiluted hemolymph was collected by removing one front fly leg at the joint nearest the thorax and then applying gentle pressure to the distal tip of the abdomen. Hemolymph exuding from the wound was collected using a glass micro-pipette and placed into a microfuge tube on ice. Hemocytes were quantified microscopically using a Bright-Line hemocytometer, and hemocyte numbers are represented as cells per µl of hemolymph.

When hemocytes were required for microscopic visualization, hemolymph was collected by employing a modified version of the high injection/recovery method previously developed for use in mosquitoes [56]. In brief, tsetse flies were sedated on ice and injected with 25 µl of chilled anticoagulant buffer [70% MM medium, 30% anticoagulant citrate buffer (98 mM NaOH, 186 mM NaCl, 1.7 mM EDTA, and 41 mM citric acid, buffer pH 4.5), vol/vol] between the last two abdominal schlerites using a glass needle and a Narashige IM300 micro-injector. Following a 30 min incubation on ice, a front leg was removed at the joint most proximal to the thorax. At this point internal pressure forced hemolymph diluted with anticoagulant buffer to be expelled from the wound site (more liquid could be recovered by applying gentle pressure to the distal end of the abdomen). Liquid was collected using a pipette and either placed into a chilled microfuge tube or directly into a 24-well cell culture plate. In the latter case, cells were allowed to adhere to the plate bottom, after which anticoagulant buffer was replaced with MM media.

Sessile hemocytes were observed by intra-thoracically injecting young and mature *Gmm* ^WT^, and mature *Gmm^Wgm^* ^−^ (*n* = 5 of each strain), with 2 µl of blue fluorescent (365/415 nm) 0.2 µm carboxylate-modified beads (Invitrogen corp.). Prior to use, beads were washed once in PBS and resuspended in 100% of their original volume. Flies were dissected 12 h post-injection to reveal their dorsal vessel and surrounding tissue, which was gently washed 3 times with PBS to remove any potentially contaminating circulating (non-adherent) hemocytes. Engulfed microspheres were visualized using a Zeiss steriomicroscope (Discovery v8) equipped with a coaxial fluorescence module. Semi-quantitative comparison of sessile hemocyte number between young and mature *Gmm* ^WT^ adults, and mature *Gmm* ^WT^ and *Gmm^Wgm^* ^−^ adults, was performed by quantifying fluorescent signal intensity (*n* = 4 individuals from each group) using ImageJ software (http://rsbweb.nih.gov/ij/).

### Phagocytosis and Melanization Assays

Phagocytosis by circulating tsetse hemocytes was observed by intra-thoracically infecting mature *Gmm* ^WT^ flies (*n* = 10) with 1×10^6^ GFP-expressing *E. coli* K12. Twelve hours post-infection, hemolymph was collected and hemocytes monitored to determine if they had engulfed the GFP-expressing bacterial cells. Hemolymph samples were fixed on glass microscope slides via a 2 min incubation in 2% PFA. Prior to visualization using a Zeiss Axioscope microscope, slides were overlayed with VectaShield hard set mounting medium containing DAPI (Vector Laboratories, Burlingame, CA).

Phagocytosis by tsetse hemocytes was inhibited with blue fluorescent (365/415 nm) 0.2 µm carboxylate-modified beads (Invitrogen Corp.). Prior to use, beads were washed once in PBS and resuspended in 100% of their original volume. Inhibition assays were performed by inoculating 8-d-old *Gmm* ^WT^ with 2 µl of beads via their thoracic compartment. Twelve hours later, these flies were similarly infected with 1×10^3^ and 1×10^6^ GFP-expressing *E. coli* K12 (experiment was performed in triplicate; *n* = 25 flies per replicate). Finally, 12 hours post-infection with *E. coli*, hemolymph was collected and processed as described above (these samples were overlayed with VectaShield hard set mounting medium that lacked DAPI).

Melanization assays were performed by intra-thoracically inoculating mature *Gmm* ^WT^ and *Gmm^Wgm^* ^−^ (*n* = 10 of each strain) with 1×10^3^ *E. coli* K12. Subsequently, three individuals from each group were monitored microscopically every 10 min for the presence of melanin at the wound site. The remaining seven flies from each group were maintained for 2 wk in order to observe infection outcome.

### Statistics

Statistical significance of survival curves was determined by log-rank analysis using JMP (v8.02) software (www.jmp.com). Statistical analysis of qPCR data was performed by Student's *t* test using JMP (v8.02) software. Statistical significance between various treatments, and treatments and controls, is indicated in corresponding figure legends.

## Supporting Information

Table S1 Primers used for quantitative real-time PCR.
(0.02 MB XLS)

## Abbreviations

AMP: antimicrobial protein
CFU: colony-forming units
dpi: days post-infection
GFP: green fluorescent protein
*Gmm^Wgm^*^−^: *Wigglesworthi*a-free *Glossina morsitans morsitans*
*Gmm*^WT^: wild-type *Glossina morsitans morsitans*
*Gmm*^WT/*Wgm*−^: adult wild-type *Glossina morsitans morsitans* treated with tetracycline
hpi: hours post-infection
iNOS: inducible nitric oxide synthase
PPO: prophenoloxidase
qPCR: real-time quantitative PCR
rRNA: ribosomal RNA
TEP: thioester-containing protein
